# A Perspective of Immunotherapy for Prostate Cancer

**DOI:** 10.3390/cancers8070064

**Published:** 2016-07-07

**Authors:** Ida Silvestri, Susanna Cattarino, Sabrina Giantulli, Cristina Nazzari, Giulia Collalti, Alessandro Sciarra

**Affiliations:** 1 Department of Molecular Medicine, Sapienza University of Rome, Rome 00161, Italy; sabrinagiantulli@alice.it; 2 Department of Urology, Sapienza University of Rome, Rome 00161, Italy; susanna.cattarino@uniroma1.it (S.C.); sciarra.md@libero.it (A.S.); 3 Department of Public Health hand Infectious Diseases, “Sapienza” University of Rome, Rome 00185, Italy; cristina.nazzari@uniroma1.it; 4 Medicine of Systems, Rheumatology, Allergology and Clinical Immunology, Translational Medicine of the University Tor Vergata, Rome 00133, Italy; giulia.collalti86@gmail.com

**Keywords:** prostate cancer, immune system, regulatory T cells, myeloid-derived suppressor cells, tumor associated macrophages, dendritic cells, immune therapy

## Abstract

In cancer patients, the immune system is often altered with an excess of inhibitory factors, such as immunosuppressive cytokines, produced by regulatory T cells (Treg) or myeloid-derived suppressor cells (MDSC). The manipulation of the immune system has emerged as one of new promising therapies for cancer treatment, and also represents an attractive strategy to control prostate cancer (PCa). Therapeutic cancer vaccines and immune checkpoint inhibitors have been the most investigated in clinical trials. Many trials are ongoing to define the effects of immune therapy with established treatments: androgen deprivation therapy (ADT) and chemotherapy (CT) or radiotherapy (RT). This article discusses some of these approaches in the context of future treatments for PCa.

## 1. Introduction

Prostate cancer (PCa) continues to be a major hurdle, as it is the second most common cancer among men. According to the National Cancer Institute, there are estimated 137.9 new cases per 100,000 men per year, which accounts for one in five new diagnoses [1]. PCa is the second leading cause of cancer-related mortality among men associated with an estimated 27,540 deaths in 2015 [2].

Patients with localized PCa are often successfully treated via local therapies (surgery or radiotherapy) [3] and patients affected by metastatic PCa received androgen deprivation therapy (ADT), which still represents the most common form of treatment [3]. When patients develop a disease state known as castration-resistant prostate cancer (CRPC), which can include various clinical states ranging from asymptomatic or minimally symptomatic, non-metastatic disease to symptomatic, metastatic diseases (mCRPC), the time of progression may vary for each patient.

For this setting of patients, following docetaxel-based chemotherapy, the FDA recently approved four new agents for the treatment of PCa: cabazitaxel, a taxane chemotherapy agent; abiraterone [4] and enzalutamide [5], which target the androgen receptor (AR) axis [6]; and radium-223, an α-emitting radiopharmaceutical [7]. However, several patients have shown primary resistance to these agents, although the mechanisms of resistance are not fully understood [8].

Therefore, additional treatment strategies are needed to further improve the survival outcomes of patients with advanced and metastatic PCa [9]. The manipulation of the immune system represents a promising approach for cancer treatment therapies, and also an attractive strategy to control prostate cancer [10,11].

This article reviews the role of immune-based therapies that target both lymphoid as well as myeloid cells and vaccines for the treatment of PCa.

## 2. Introduction to Immunotherapy

Many studies have reported a continued interplay between tumor cells and the microenvironment that may prove decisive in disease outcomes [12]. Host antitumor reactions could be considered two sides of the same coin in terms of their consequences in tumor development. Persistent inflammatory reactions may be an important contributor to tumor progression, whereas immune response to tumor cells may inhibit disease progression [13].

Furthermore, several pieces of evidence have indicated that the tumor microenvironment alters myeloid and lymphoid cells, facilitating the suppression of the host immune response [14].

Actually an immune response that is primarily cell-mediated has been observed in the normal human prostate [15]. Then histological data have revealed the presence of CD4+ T cells, CD8+ T cells, natural killer (NK) cells, dendritic cells (DC), and macrophages within tumors [16,17,18,19]. Generally, a dense infiltration of lymphocytes has been correlated with longer patient survival, whereas a significant reduction of T cells is detected in high grade prostatic adenocarcinomas compared to benign nodular prostatic hyperplasia [16,17,18,19], suggesting that tumor progression may be associated with alterations in cell-mediated immune responses [20,21]. Furthermore, high density of M2-polarized tumor-associated macrophages (TAM) is observed in both epithelial and stromal compartments and is statistically associated to poorer prognosis [22,23]. TAMs are a significant component of the inflammatory infiltrates in PCa. Moreover, increased TAMs levels in biopsy are predictive of worse recurrence free survival in men treated with primary ADT [24]. An inverse correlation between total macrophage density and time to recurrence has also been reported from different analysis [24,25]. Similar results were observed for Tregs [18,26,27,28]. Another negative prognostic factor is represented by increasing myeloid-derived suppressor cells (MDSC) detected both at tumor site as well in the peripheral blood of patients. The increase of these cells correlated with other negative prognostic factors, such as lactate dehydrogenase, alkaline phosphatase, PSA, and anemia in PCa [29]. Finally, a strong correlation between the DCs and PCa characteristics has also been observed. Patients with metastatic disease showed fewer circulating myeloid DCs than their age-matched controls [30] and a lower number of DCs was parallel with a higher Gleason score while DCs are elevated in low risk cancer [31]. These results indicate that, in PCa patients, monocytes do not develop into myeloid DCs as efficiently as they do in healthy individuals. This idea is also supported by observations that serum of PCa patients inhibited monocytes differentiation into DCs and that the degree of inhibition correlated with higher PSA levels [32].

Crosstalk between these cells could promote synergy and amplify the immune suppressive effects of individual cell population in PCa as well as in many other tumors [33].

The goal of immunotherapy is to overcome such mechanisms in order to detect and destroy cancer cells or at least to induce the pathways that go back from “the escape phase to equilibrium phase” according to the definition of the cancer immunoediting [12].

Cancer immunotherapy has recently been introduced into the therapeutic field of metastatic PCa and mCPRC. Current immunologic approaches with particular relevance to mCPRC are discussed in a more detailed way in the following sections dividing into antibodies, vaccines, adoptive T cell therapy (ADT) while in the last section several immune functions are targeted by a chemical compound.

## 3. Immune Check Point Inhibitor

The immune check points are important for allowing the immune system to maintain homeostasis and self-tolerance. This is obtained by down regulating T cell activation or effector functions [34]. However, these check points may also represent a common *mechanism* of *tumor cell escape* from the immune system [35]. There are currently several ongoing investigations but the most promising results for cancer therapy have been obtained by targeting the Cytotoxic T-lymphocyte-associated protein 4 (CTLA-4) and Programmed T cell death 1 (PD-1) receptors [34,35,36,37]. CTLA-4, expressed on activated T cells and Tregs, down-regulates the extent of T cells activation by determining the balance with CD28 signals [38]. Both CTLA-4 and CD28 bind the ligand B7-1 (CD80) and B7-2 (CD86) but CTLA-4 has a higher affinity than CD28 [39,40]. The binding with CTLA-4 limits T cells expansion by reducing the production of an important growth factor as IL-2, and also by inhibiting TCR-mediated induction and assembly of essential components of the cell cycle machinery [40]. Targeting CTLA-4 in order to remove inhibition signals for effector T cells, deplete suppressor Tregs and restore an immunological response against the tumors is a long story. It began with the first murine models in 1996 [35], passed through the first FDA approval in 2011 as a treatment for patients with advanced melanoma [41] and many clinical trials are currently ongoing on several different types of tumors [37] included PCa [42].

The other promising therapeutic strategy is directed to PD-1 and its ligands. PD-1 is an immune regulatory T cells agent that is expressed on a subset of thymic T cells; it is upregulated on activated NK, B and T cells [43]. PD-1 has two ligands: PD-L1 (B7-H1) and PD-L2 (B7-DC) that are expressed on antigen presenting cells (APC) [44]. PD-1 is normally involved in promoting tolerance and preventing tissue damage in setting of chronic inflammation [45]. The interaction PD-1 with PDL-1 inhibits T cell receptor signaling and downregulates the expression of some antiapoptotic molecules and pro-inflammatory cytokines [45]. The interaction of PD-1/PD-L1 is likely the principal mediator of immunosuppression [46].

The expression of PD-L1 on tumor cells is thought to play a role in decreasing the immune responses against the tumor contributing to tumor progression [47]. PD-L1 is expressed on a variety of solid cancers and is correlated with a worse prognosis. Moreover, an over expression of PD-1 on tumor infiltrating lymphocytes (TIL) matched with compromised antitumor response [48]. Finally, preclinical data have reported that blockading PD-1 or PD-L1 could restore immune function resulting in a reduction of tumor load and metastatic spread [48]. To date, three monoclonal antibodies (mAb) against PD-1, and one against PD-L1 have been analyzed in Phase I trials [48]. All four agents have shown encouraging preliminary activity, and those that have been evaluated in larger patient populations appear to have also an acceptable safety profile [49,50]. Using mAbs that inhibit the interaction between PD-1 and its ligand has shown the most significant antitumor effects primarily in melanoma however there are interesting prospective for other types of tumors [51].

The challenges are not always without risks and targeting immune check point increased immune surveillance but could also break immune tolerance to self and cause autoimmune side effects. Such immune-related adverse events (iAE) most commonly manifest as diarrhea, colitis, rash, and pruritus (grade 1–2) or hepatitis, hypophysitis, and thyroiditis (grade 3–4) and are generally manageable and responsive to corticosteroid therapy or other immune suppressive agents [52]. Remarkably, immunosuppressive therapy does not appear to moderate ongoing antitumor effects [53].

*Ipilimumab* (Yervoy), an anti-CTLA-4 mAb and fully human IgG1 (Bristol-Myers Squibb), was the first immune check point blocking compound to enter in oncology clinical trial. The clinical activity obtained in melanoma was very encouraging with significant improvement of overall survival (OS) among patients with metastatic melanoma [41] and manageable iAEs [41]. Long term follow-up showed that 19%–36% of patients with metastatic melanoma treated with ipilimumab had long-term survival, some with survival rates extending up to four years [54,55,56,57], besides patients non-responsive became responsive after a more long time [56]. These results have been used to evaluate the therapeutic potential in a variety of solid cancers. Ipilimumab is currently in trials for the treatment of advanced non-small cell lung cancer (NSCLC) [58], metastatic renal cancer [59] and ovarian cancer [60]. Regarding PCa, initial studies have shown that ipilimumab (3 mg/kg) administered every four weeks for a total of four doses had acceptable safety profile [42] but anticancer effects were obtained especially in combination therapy [61,62,63]. In some CRPC patients, combining CTLA-4 blockade with systemic granulocyte-macrophage colony-stimulating factor (GM-CSF) induced a decline in PSA with an expansion of activated circulating CD8+ T cells, presumably mediated by preexisting tumor-specific T cells that were primed by endogenous tumor-derived antigens and were receptive to the CTLA-4 blockade [61]. Synergic antitumor activity was evaluated also by combining immunomodulatory therapy with radiotherapy. Data showed that not only radiotherapy is not immunosuppressive but works synergistically with immunotherapy for enhancing antitumor immune responses, inhibiting immunosuppression, and/or altering tumor cell phenotype in order to increase their susceptibility to immune-mediated killing [64]. In a phase I–II study [62], ipilimumab administered alone in increasing dosage or in addition to a single dose of radiation each day was associated with clinical antitumor activity and disease control in the mCRPC patients primarily in who received ipilimumab 10 mg/kg ± radiotherapy [63]. But at high dosage (10 mg/kg) independent from radiotherapy iAEs became more frequent (all grades, 80%; grade 3/4, 32%), and in some cases last longer [62,63].

Further, a Phase III trial enrolled *docetaxel-refractory* men with mCRPC that were treated with radiation therapy to a bone metastasis followed by either ipilimumab (10 mg/kg every three weeks for a total of four doses) or placebo. In this trial, the patients lacking visceral disease and with favorable laboratory values treated with ipilimumab reported a prolonged OS [64,65].

Ongoing clinical trial is combining ipilimumab (3 mg/kg) and degarelix (a gonadotropin-releasing hormone (GnRH) receptor antagonist). This study provides two cohorts: one uses ipilimumab and degarelix prior to and following radical prostatectomy in men with newly mCRPC and the second cohort includes men who have already received definitive local therapy with radical prostatectomy but have since experienced biochemical and/or metastatic recurrence (NCT02020070). An overview of relevant ongoing clinical trials is provided in Table 1.

***Tremelimumab***, a fully human IgG2 (Pfizer, MedImmune) (CP-675,206) has been investigated in PCa both in neoadjuvant and in recurrent disease. In particular, in a phase I dose escalation trial [66], tremelimumab was combined with short-term ADT in patients with PSA-recurrent PCa. Three of 11 patients reported a prolongation in PSA doubling time detectable only several months after completing treatment while no favorable changes in PSA doubling time were observed in a shorter periods after completing treatment [66].

Tremelimumab was also included in a multicenter Phase I–II study combined with the PD-L1 antibody, durvalumab (MEDI4736), and the tumor microenvironment modulator polyICLC, a Toll like receptor-3 (TLR-3) agonist, in patients with several advanced, measurable, biopsy-accessible of cancers, including PCa (NCT02643303).

*Nivolumab* (Opdivo) an anti PD-1 mAb and fully human IgG4, approved by the FDA in 2014 for the treatment of malignant melanoma progressing after treatment with ipililumab or after treatment with serine/threonine protein kinase B-Raf (BRAF) inhibitor [67,68,69,70]. Moreover nivolumab was also approved in 2015 for the treatment of refractory squamous NSCLC [70]. Recently, in December 2015, the FDA expanded the label to include the approval of Pembrolizumab (Keytruda) [71] for patients with metastatic melanoma who had not received prior ipilimumab.

A phase I study was performed to evaluate nivolumab in patients with a variety of malignancies [70]. This trial showed a favorable safety profile and a preliminary evidence of clinical activity, primarily in melanoma patients. In PCa, immune-histochemical analyses of PD-L1 expression have suggested an association of PD-L1 with a more aggressive tumor, indicating that the PD-1/PD-L1 pathway is correlated with the lower of antitumor immune response, promoting tumor proliferation and progression [72]. Further studies reported simultaneous high PD-1/PD-L1 expression in PCa patients enrolled to receive targeted anti-PD-1/PDL1 immunotherapy [73]. However only one of 17 patients with CRPC enrolled in the nivolumab trial reported a 28% reduction in measurable lesions and therefore no objective responses [74].

An approach to improve cancer immunotherapy has combined the two check point modulators. Indeed, ipilimumab and nivolumab have different biological characteristics that could result in synergic antitumor activity. Combining ipilimumab and nivolumab was analyzed in patients with metastatic melanoma to detect the maximum tolerated dose with the best clinical response [75]. The first clinical results reported that the two immune check points work better in combination than when given separately. The melanoma patients experienced greater decrease in tumor size with longer progression-free survival (PFS) than either compound alone [76,77].

Combined trial is ongoing also in PCa, a phase II clinical trial (NCT02601014) is currently enrolling mCRPC patients with detectable androgen receptor-variant-7 (AR-V7), (tumor cells expressing AR-V7 has been shown to be resistant to hormone therapy and some chemotherapies). The outcome measures include a decline in PSA and PFS.

## 4. Therapeutic Cancer Vaccines

Therapeutic cancer vaccines have emerged as an attractive strategy to induce an antitumor immune response to shrink tumor and to protect against tumor recurrence or metastatic disease [78]. Preparing a successful cancer vaccine requires the selection of the opportune antigens and the suitable adjuvants to restore the immune response against the tumor, as well as the administration route [79,80]. Moreover, because immune responses may depend on presentation of the vaccine antigens by DCs, it is advantageous to administer APCs (i.e., DCs) with tumor antigens [79,80]. DCs have a critical role in preparation of vaccine, are routinely prepared ex vivo from peripheral blood mononuclear cells (PBMC) leukapheresis or buffy coats from CD34+ progenitor cells or CD14+ monocytes. Mature and immature DCs have been used, but mature DCs are superior in the induction of immune responses while immature DCs may induce tolerogenic responses [81]. The prostate represents an ideal target for cancer vaccines. Indeed several prostate-specific proteins have been identified to induce an immune response. In PCa, the immune response to specific antigens was approached by using both autologous and allogenic strategies [10,82,83].

***Sipuleucel-T*** (Provenge) was the first autologous cellular immunotherapy approved by the FDA in 2010 and by the European Medicines Agency (EMA) for the treatment of asymptomatic or minimally symptomatic mCRPC [84], and to date it remains the only FDA-approved immunotherapy for PCa. This vaccine targets prostatic acid phosphatase (PAP), a secreted glycoprotein enzyme synthesized in prostate epithelium that significantly increases as cancer progresses; it is elevated in patients with bone metastasis and is associated with responsiveness to therapy and a shortened survival [85]. Rodent model and preclinical studies have determined that PAP elicits PAP-specific humoral and cellular immunity [86]. GM-CSF was added to stimulate APCs and obtain more mature DCs [87]. Autologous APC-containing peripheral blood mononuclear cells (PBMC) of PCa patients were harvested from a leukapheresis procedure, then transferred in a facility and incubated ex vivo for 36/48 h with a fusion protein (PA2024) combining PAP and GM-CSF. The fusion protein was washed out and finally the product was reinfused into the patient. This product contains at least 5 × 10^7^ autologous activated CD54+ DCs and a variable number of T cells, B cells, NK cells, and other cells [87,88]. APCs should process the recombinant target antigen PAP-GM-CSF into small peptides that are presented to T cells [81].

Sipuleucel-T was routinely administered to patients via intravenous infusion. In a full course of therapy, this process is repeated twice at approximately two-week intervals. Phase I–II clinical trials have shown a good response to sipuleucel-T with few side effects that were primarily transient, low grade and infusion related [89,90]. After the treatment, the patients developed an appreciable antigen specific T-cells activation and a production of antibodies against the fusion protein. Moreover a PSA decline of more than 50% was reported in approximately 10% of patients [89,90].

Three phase III clinical trials have been completed. The two first studies analyzed sipuleucel-T versus placebo in asymptomatic mCRPC patients. The time to progression did not differ but there was a significant increase of OS (25.9 months versus 21.4 and 19.0 months versus 15.7) in patients treated with Sipuleucel-T [87,91]. The Phase III clinical trial, known as the Immunotherapy for Prostate Adenocarcinoma Treatment (IMPACT), enrolled asymptomatic and symptomatic or minimally symptomatic mCRPC patients and reported a 4.1 months improvement in the median OS and at 36 months the survival rate was 31.7% for treated patients compared to 23.0% for patients treated with the placebo [92], no significant difference in the time to disease progression (14.6 weeks vs. 14.4 weeks). Data from the IMPACT study showed that the greatest benefit occurred in patients with a lower disease burden [92,93]. Studies addressed for evaluating immune responses showed that sipuleucel-T induces the antigen-specific immune response as well as promotes the recruitment of activated effector T cells in the prostate tumor microenvironment [94,95]. Early screening and diagnosis are important for identifying patients who may benefit most from sipuleucel-T treatment. Patients with lower PSA levels indicative of an early stage of mCRPC appear to get better clinical outcomes [95,96]. Many studies are aimed at clarifying the mechanism of action of sipuleucel-T, at the identification of new biomarkers to predict survival benefit and at optimizing the doses and the schedules of treatment of sipuleucel-T when combined with other therapies in clinical [95,96].

In fact this treatment has short duration (~4 week), providing an opportunity for patients to receive subsequent treatment. Most patients in the IMPACT study went on to receive other therapies [83]. Sipuleucel-T is also trialed combined with chemotherapy. Experimental data obtained in mice and humans have also contradicted the traditional thinking that taxanes suppress immune-cell functions. The same concepts are for radiotherapy [88].

A recent study has evaluated abiraterone in combination with sipuleucel-T and no alteration in immune parameters that correlates with sipuleucel-T was detected. A long-term follow-up for OS is ongoing in the STAMP study [97].

A case report described that the treatment with sipuleucel-T following to enzalutamide in a patient with mCRPC resulted in a complete and durable biochemical response [98].

Another approach of combining therapy with activated DCs is DCVAC/PCa.

*DCVAC/PCa* is an autologous immunotherapy. Immature DCs were harvested from leukapheresis and pulsed with killed LnCaP, a PSA-positive prostate cancer cell line. Tumor cell-pulsed DCs were then matured with 25 μg/mL of Poly I:C. Such vaccine was injected in patients with mCRPC that have already received docetaxel. From this Phase I/II clinical trial is resulted that the therapy was well tolerated and iAEs were low grade [99]. Actually, a phase III clinical trial (NCT02111577) is ongoing to evaluate the efficacy and the safety of DCVAC/PCa versus placebo in men with mCRPC eligible for first line chemotherapy. Lastly, a similar approach is in BDCA/1 BDC-01.

*BDCA/1 BDC-01* is an autologous vaccine prepared by pulsing autologous CD1c+ BDC from leukapheresed PBMCs with a cocktail of HLA-A*0201-restricted peptides (PSA, PAP, prostate specific membrane antigen, and control influenza peptide) and keyhole limpet hemocyanin [100]. It was injected in 12 patients with mCRPC. Until now, no acceleration of disease was noted, PSA levels remained unchanged and iAEs were of low grade [100]. Other trials are necessity to evaluate its efficacy.

*Prostvac-VF* (viral-based vaccine) is a recombinant viral vaccine currently studied as an immunotherapy for PCa. Prostat-VF (TRICOM or PSA TRICOM) is based on a combination of two viral particles, vaccinia that is a potent immunologic priming agent, followed by fowlpox that is minimally or nonreactive to vaccinia that is used as a boosting agent [101]. Prostvac-VF targets PSA, one of the first antigens discovered to be expressed at substantial levels by most patients with PCa. It is known that PSA is released by the ductal and acinar epithelial cells of the prostate gland in males [85].

Both recombinant viruses were engineered to encode the entire PSA gene with a modified agonist epitope [101,102,103]. In addition, DNA encoding costimulatory molecules was incorporated in order to further enhance the immune response: B7-1 (facilitates T cell activation), lymphocyte function-associated antigen 3 (LFA-3; CD58, enhances signaling through the T cells receptor for antigen), and intercellular adhesion molecule-1 (ICAM-1; CD54, a cell surface adhesion molecule which plays a prominent role in regulating the migration and activation of both DCs and T cells) [101,103]. APCs should be activated directly by viral vectors and mainly by cellular debris containing encoded antigens derived from infected epithelial cells. Activated APCs present antigens to CD4+ and CD8+T cells and induce T cell mediated immune response that detect and destroy PSA expressing cells [101,104].

Prostvac-VF does not require complex individualized therapy and is manufactured and reproduced easily. Negligible side effects were observed in treated patients; most iAEs were injection site related, that were identified in only a subset of patients and associated with symptoms of fatigue fevers, and nausea (grade 2 toxicity) and some flu-like symptoms [101].

Prostvac-VF was evaluated in a randomized phase II clinical trial in men with mCPRC. Comparing men who received Prostvac-VF and GM-CSF with men who received an empty vector with a placebo, the study showed positive results in the median OS with a difference of eight months in the treatment groups. The medians OS for the control group was 16.6 compared to 25.1 months for the Prostvac-VF group [105]. Additionally, an increase in T cell response, greater than six-fold, and a lower Tregs was observed in patients who survived longer than expected according the Halabi prognostic model. No difference was detected for the GM-CSF combination [106]. In addition, for Prostvac-VF treatment, patients with less aggressive or early stage disease exhibited greater benefits [105,106].

A study addressed to understand the impact of this vaccine on generating tumor-specific T cells determined that a T-cell response, mostly CD8^+^, appeared predominant, with no evidence of B-cell response. This is a promising data considering the depletion of CD8^+^ at prostate cancer sites. Furthermore, the absence of antibody against PSA allows for the use of PSA levels to assess disease kinetics in vaccine-treated patients [107]. 

An international phase III trial of asymptomatic or minimally symptomatic men with mCRPC for treatment with and without GM-CSF is currently ongoing (NCT01322490). More than this an open label, randomized trial of PROSTVAC in combination of PROSTVAC with ipilimumab in localized PCa (NCT02506114).

*GVAX*, granulocyte-macrophage colony-stimulating factor tumor cell vaccine, represents a whole-cell based immunotherapy. In this approach, whole autologous or allogeneic tumor cells as source of immunogens are genetically modified to express GM-CSF. This growth factor induces an advantageous microenvironment for tumor antigen presentation through the recruitment of APCs, a critical step in the induction of an optimal immune response to any immunotherapy [10]. Because the small number of cells that can be obtained from surgically removed tumors limits autologous approach, GVAX for PCa is composed of two human prostate cell lines, LnCaP (androgen sensitive derived from a lymph node metastasis) and PC3 (androgen insensitive derived from bone metastasis) as antigens source, which are transfected with GM-CSF, and then irradiated for safety [107,108]. Phase I–II trials enrolling patients with CRPC, chemotherapy-naive, received an intradermal priming vaccination with GVAX-PCa (5 × 10^8^ cells, half quantity of each cell line) followed by 12 weekly boost for six months [107] or ranged doses (1 × 10^8^ cells to 5 × 10^8^ cells) [108]. This immunotherapy was well tolerated and immunogenic for several patients in terms of dose and time treatment and was associated with an encouraging OS rates. These data supported to perform two phase III trials to confirm the survival benefits. The first phase III trial, Vaccine Immunotherapy with Allogeneic Prostate Cancer Cell Lines (VITAL)-1, was designed to compare GVAX to docetaxel plus prednisone in asymptomatic CRPC [109,110]. VITAL-2 was conducted in symptomatic CRPC patients [109,110]. The VITAL-2 study was early stopped due to increased mortality in the vaccine arm. The VITAL-1 study was also stopped based on a futility analysis because its primary end point was indicated as less than a 30% [109].

Preclinical studies have shown that GVAX combined with ipilimumab has a potent synergic effect evidenced by both biochemical and radiological responses in mCRPC patient [110]. The safety profile resulted in this study warrant further research.

## 5. Adoptive T Cell Therapy

Another strategy to induce the immune response against the tumor is directed to the adoptive T cell therapy (ACT). This therapy involves the isolation and the expansion of T cells, activated ex vivo, then reinfused into cancer patients with the goal of recognizing, targeting, and destroying tumor cells [111]. 

It has long been known that ACT based on tumor-infiltrating lymphocytes (TILs) can induce tumor regression [112]. Currently the best clinical responses are achieved in patients with metastatic melanoma [112]. Some treatments, such as lymphodepletion prior the reinfusion of TILs further enhance the responsivity [113]. The optimization of the treatment required also the co-administered of high dose of IL-2, which on the other hand can cause toxicity [114]. Probably, these conditions by removing Tregs and by stimulating T cells can overcome immune suppressive microenvironment [115] and mediate tumor regression, as observed in 50%–70% of patients with metastatic melanoma [116].

TILs can be grown from several cancer types, such as kidney, breast and colon but until now they have not shown cytolytic activity against autologous tumor cells [117]. Ongoing studies are addressed to other cancers that could express targeting antigens to TILs. Somatic mutations are often detected in tobacco related tumors, such as lung carcinomas and head and neck cancer [117]. Furthermore viral proteins expressed in human papilloma virus (HPV) primarily associated to oropharyngeal and cervical cancers, can be the targets of TILs, as well as Epstein-Barr viral (EBV) proteins expressed in EBV-related cancers, included most Burkitt’s lymphoma, undifferentiated nasopharyngeal carcinoma, and lymphoproliferative disorders, some Hodgkin’s disease and non-Hodgkin’s lymphoma, and gastrointestinal lymphoma [117].

Since TILs with tumor-specific receptors can only be generated from some cancer patients, ADT has been improved by introducing antigen receptors into circulating lymphocytes.

*CAR T cells*, chimeric antigen receptors T cells, represent a therapy based on engineering patient’s T cells, specifically modified to recognize and destroy the tumor cells [118]. T cells can be reprogrammed to express chimeric antigen receptors (CAR) so that the specificity of the antibodies are combined with the cytotoxic functions of T cells in order to target several tumor antigens [119]. As above describe, PBMC must first be collected by leukapheresis and grown under conditions that will support the expansion and the stimulation of T cells.

CARs are generally composed of an extracellular single-chain antibody variable fragment (scFv) directed to TAA that is linked, via hinge and transmembrane domains, to intracellular signaling domains [120,121]. Genetic material is transferred into the patient’s T cells using either viral or non-viral vectors [121,122]. Because they are derived from antibodies, the recognition of target TAAs is not MHC-restricted and this technique can be applied to all individuals irrespective of their HLA type, and it can also recognize carbohydrate and glycolipid antigens.

Currently three generations of CARs have been described. First-generation contained only T cell CD3ζ chain and antigen recognition domains while subsequent second- and third-generation additional costimulatory molecules, such as: CD28, 4-1BB, CD27, ICOS or OX40, were added to increase the antitumor effects and to improve the proliferation and the survival of CAR T cells [123,124,125,126,127].

The clinical trials using first generation CAR T cells reported no objective responses [111]. Afterwards, the most clinical positive data have been achieved in the treatment of hematological malignancies [128], particularly with CD19-specific CAR T cells in patients with relapsed or refractory B-cell malignancies [129,130,131,132]. These results have raised strong interest in development of CARs for solid tumors as well and many studies are rapidly evolving in order to design an optimal CAR for the best results in human trials.

Indeed, upon infusion into a patient, CAR T cells must overcome a number of obstacles before attacking tumor cells and for this reason many strategies are still under investigation to improve the different steps.

Critical step is to determine the conditions leading to an optimal CAR T cells persistence [133] and selectively reducing Tregs [134]. It is also important to define those cytokines to include into CAR T cell gene in order to induce the physiologic mechanisms, memory formation and antigen-driven expansion [135]. Additionally, because CAR T cells are “living drugs” some imperfection may result in T cells that target and damage undesirable tissue or in over-proliferation. Thus, suicide genes are also necessary [136,137]. In solid tumors, other critical points are represented from tumor vasculatures, vascular normalization resulted after the treatment with low doses of angiogenesis inhibitor improved CAR T cells response [138,139]. As well as engineering with ligands of specific chemokines allows CAR T cells to more easily reach primary tumor and specially the metastasis [140,141]. Studies are also aimed to ameliorate CAR using humanized antibodies binding multiple antigens [142,143].

Until now, the most relevant results come from clinical studies with such therapy based on anti-CD19 CARs against a variety of B-cell malignancies. Treatment is associated with transient but frequently severe iAE related to elevated serum cytokine levels [117].

The greatest challenge is the identification of antigens in solid tumors target for CAR T cells therapy. Many tumor cell surface antigens are also expressed at low level on healthy tissue so that an immune response could be activated against vital organs. Mesothelin [144] and Epidermal growth Factor variant III (EGFvIII) [145,146] are presently being studied in a phase I clinical trial with careful dose escalation [117].

In PCa, CAR T cells can be engineered to target PSCA and PSMA. Slovin et al have established an ex vivo transduction, expansion and therapeutic protocol for the generation and to testing the safety, clinical-grade, PSMA targeted T cells in PCa [147].

There are only few clinical trials, and thus much more research is needed. For most patients, iAE are mild enough and can be managed with standard supportive therapies, including steroids. Cytokine-release syndrome is the most troublesome effect also induced in patients treated with Car T cells.

## 6. Tasquinimod

One compound under clinical investigation for the treatment of PCa as well as several solid tumors is tasquinimod (TasQ) [148]. Chemically, it is a second-generation quinoline-3-carboxamide compound with lack of pro-inflammatory effects and high efficacy against antiangiogenic activity [149]. The antiangiogenic property of this molecule is important because tumor growth inhibition induced by TasQ was associated with reduced microvasculature density, increased expression, and secretion of the angiogenesis inhibitor thrombospondin-1 (TSP-1), and downregulation of VEGF and hypoxia-inducible factor-1α (HIF) [150]. 

This orally agent has shown efficacy and favorable safety profile in Phase I–II clinical trial in PCa. At present time, phase III clinical trial on CRPC patients is in progress. Preclinical studies show that TasQ suppresses reciprocal cross-talk between cancer and tumor infiltrating host cells such as endothelial cells, MDSC, and macrophages [151,152] contributing to its pleiotropic effects, including anti-angiogenesis, immunomodulation, and inhibition of metastasis [153,154]. Although its mechanism of action is still under investigation, some target results seem to have an important role.

In this regard, the calcium binding protein, S100A9 has been identified as a potential target of TasQ [155,156]. S100A9 interacts with pro-inflammatory receptors: Toll like receptor (TLR)-4 and receptor of advanced glycation end products (RAGE) [155,156,157]. These receptors are expressed on MDSC and S100A9 regulates the recruitment from bone marrow [158] and the accumulation of these cells in tumor microenvironment inhibits the immune response [33]. The modulation of MDSCs represents a critical biologic mechanism of action of TasQ, indeed MDSCs induce an immune suppressive microenvironment and promote the M2-polarized TAMs that support angiogenesis and metastasis [33,159,160]. In prostate cancer, S100A9 is upregulated in both prostatic intraepithelial neoplasia and adenocarcinoma, whereas benign prostatic tissue showed minimal to no expression [161]. It is probably that antitumor activity of TasQ, in prostate as well as in other tumors, is attributable to the suppression of MDSCs [157].

In addition, results from other studies have reported histone deacetylase (HDAC)-4 as a molecular target for TasQ. TasQ binds and blocks HDAC4 in an inactive conformation, preventing epigenetic reprogramming needed for the angiogenic switch induced by HIF-1α [162]. HDAC-4 is overexpressed in CRPC and in vitro studies have shown a growth inhibition induced by suppression of HDAC-4 [162].

At dosage of 0.5–1 mg per day, TasQ is well tolerated and only common iAEs are reported in a phase I trial. A phase II trial, TasQ doubled the PFS at six months and prolonged survival of patients with mCRPC compared to placebo [152,163,164]. Exploratory biomarker analyses indicate that TasQ treatment had a more pronounced effect on OS in men with low levels of biomarkers, indicating a greater impact in men with a lower disease burden, similar to the observations for other immunotherapies. The systemic TSP-1 levels below the median at baseline correlated with a survival benefit with TasQ versus placebo [164]. A subsequent analysis of bone scan index (BSI) suggested a modest, short-term delay in objective radiographic bone scan progression [165]. Based on these preclinical and clinical observations, a phase III double-blind, placebo-controlled international trial of men with mCRPC and bone metastases was conducted (NCT01234311). Although the final manuscript and presentation are not yet available, the effects of TasQ are not consistent with the phase II observations [166]. It may be that TasQ, similar to other compounds, as a single agent has a modest activity, and only in combination can improve clinical outcome.

In this regards, a phase I non-randomized trial study is focused on evaluating in mCRPC men with chemorefractory the combination of prednisone and cabazitaxel with TasQ at the administration of 0.25 mg, 0.5 mg, and 1.0 mg (NCT01513733).

## 7. Conclusions

Immunotherapy can trigger a dynamic immune response that can kill tumor cells for an extended period time. Progress in immunotherapy has been a result of significant advances in our understanding of the complex nature of the regulatory events in cytotoxic T cell-mediated immune responses, particularly antigen presentation, activation and immuno-editing in the cancer microenvironment. Despite the success of these agents, their efficacies do not appear to be equal for all solid tumors.

Substantial evidence has suggested that combining different immunotherapeutic approaches, as well as combining other local or systemic cancer therapies, may likely be required to realize synergistic benefits. This could be a daunting task, given the non-classic cancer responses to immunotherapy and co-evolving immune escape mechanisms. Knowledge-based trials to help inform dosing, timing, and sequencing and the development of precise criteria for patient selection are needed. Studies regarding possible combinations of immunotherapies are ongoing to better establish safety and toxicity in addition to the efficacy of such treatments. We believe that identifying the doses and timing and sequences of combined treatments are crucial for gaining synergic effects.

## Figures and Tables

**Table 1:** An overview of relevant ongoing clinical trials in PCa.

Therapy	Molecule	Mechanism of Action	Clinical Trial	Trial Identifier
*Ipilimumab* (Yervoy^®^)	IgG1 Human monoclonal antibody	Blocks the activity of CTLA-4 and T reg expression	Phase II	NCT01377389
			Phase I–II	NCT01688492
			Phase II	NCT01498978
			Phase II	NCT02113657
			Phase I	NCT00064129
			Phase II	NCT02020070
			Phase II	NCT02423928
			Phase II	NCT02279862
*Tremelimumab* (CP-675,206)	IgG2 Human monoclonal antibody	Blocks the activity of CTLA-4 and T reg expression	Phase I	NCT02643303
			Phase I	NCT02616185
*Nivolumab* (Opdivo^®^)	IgG4 Human monoclonal antibody	Blocks the activity of PD-1	Phase II	NCT02601014
*Sipuleucel-T* (Provenge^®^)	Autologous cellular immune-therapy	Stimulates a T cell immune response against cancer cells	Phase II	NCT01706458
			Phase II	NCT02159950
			Phase II	NCT01560923
			Phase I	NCT01832870
			Phase I	NCT02036918
			Phase II	NCT01807065
			Phase II	NCT02232230
			Phase II	NCT01818986
			Phase II	NCT02463799
			Phase II	NCT01420965
			Phase II	NCT01804465
			Phase II	NCT01881867
			Phase II	NCT01487863
			Phase II	NCT01981122
*DCVAC/PCa*	Autologous Dcs pulsed with killed PSA- positive LnCap cells	Evocation of immune response	Phase III	NCT02111577
*Prostvac-VF*	Viral based vaccine	A recombinant viral vaccine based on combination of two viral particles. Promotes an immune response against PSA expressing cells	Phase III	NCT01322490
			Phase II	NCT02153918
			Phase II	NCT01341652
			Phase II	NCT00450463
			Phase II	NCT01875250
			Phase II	NCT02326805
			Phase II	NCT02506114
*GVAX*	Granulocyte-macrophage-colony stimulating factor tumor cell vaccine	Evocation of a strong immunoreaction by antigens expressed on human prostate cell lines modified by GM-CSF	Phase I–II	NCT01696877
*CAR T cells therapy*	Chimeric antigen receptor T cells	Engineered patient’s T cells modified to recognize and destroy tumor cells	Phase I	NCT01140373
*Tasquinimod* (ABR-215050)	Quinolone-3-carboxamide analog	Inhibition of angiogenesis and immunomodulation	Phase I	NCT01513733
